# Human epidermal growth factor receptor 2 (HER2) immunoreactivity: specificity of three pharmacodiagnostic antibodies

**DOI:** 10.1111/j.1365-2559.2011.04034.x

**Published:** 2011-11

**Authors:** Anne-Sofie Schrohl, Hans Christian Pedersen, Sussie Steen Jensen, Signe Lykke Nielsen, Nils Brünner

**Affiliations:** The Faculty of Life Sciences, Institute for Veterinary Disease Biology and Sino-Danish Breast Cancer Research Centre, University of CopenhagenFrederiksberg, Denmark; 1 Dako Denmark A/SGlostrup, Denmark

**Keywords:** antibody specificity, HER2 protein, immunohistochemistry

## Abstract

**Aims:**

The availability of specific antibody-based test systems is essential to testing of HER2 protein expression. Here, we mapped epitopes recognized by three pharmacodiagnostic HER2 antibodies and investigated their specificity towards peptides and fusion proteins homologous to the intracellular domains of HER1, HER2, HER3 and HER4. The investigated antibodies were PATHWAY® HER2 (clone 4B5; Ventana Medical Systems Inc., Tucson, AZ, USA), HercepTest™ (Dako Denmark A/S, Glostrup, Denmark), and Oracle® HER2 (clone CB11; Leica Microsystems GmbH, Wetzlar, Germany).

**Methods and results:**

Epitopes were mapped using the alanine scanning method. Specificity was investigated in immunohistochemical stainings, competitive enzyme-linked immunosorbent assay (ELISA) and immunoblotting. All three antibodies reacted with HER2 proteins and peptides in immunohistochemical stainings, ELISA and immunoblotting. PATHWAY® HER2 also stained HER4-expressing cells, reacted with HER4 peptide in ELISA and detected HER4 fusion protein in an immunoblot. Oracle® HER2 weakly detected HER4 in immunohistochemical stainings, whereas the HercepTest™ antibody showed no cross-reactivity with other HER proteins.

**Conclusion:**

Our study shows that the PATHWAY® HER2 antibody can bind HER4 peptides and fusion proteins in three different experimental settings. This should be investigated further to determine whether binding of HER4 also occurs in tissue samples and if such binding would have implications for therapy decisions for breast cancer patients.

## Introduction

Human epidermal growth factor receptor 2 (HER2) belongs to the family of epidermal growth factor receptors (EGFRs). This family consists of four members; epidermal growth factor receptor (EGFR), HER1, HER2, HER3 and HER4. HER proteins show extensive sequence homology and through formation of homo- and heterodimers induce complex intracellular signalling (reviewed in Yarden and Sliwkowski^1^).

HER2 protein is overexpressed in approximately 20–25% of breast tumours and overexpression correlates with amplification of the *HER2* gene.^2^,^3^ Overexpression of HER2 protein and/or amplification of the *HER2* gene are associated with a poor outcome in breast cancer patients.^4^,^5^ Expression of HER1, HER3 and HER4 in breast tumour tissue has also been demonstrated; however, the reported fraction of tumours expressing or overexpressing these HER proteins vary.^6^–^8^ Expression of HER1 and HER3 has been linked with a poor outcome and increased cell proliferation in breast cancer, whereas HER4 expression has been associated with reduced mortality and decreased proliferation.^6^–^8^

Breast cancer patients whose tumours overexpress HER2 and/or show amplification of the *HER2* gene are candidates for HER2-targeted therapy with trastuzumab^9^ or other HER2-targeting drugs. Testing of HER2 protein expression by immunohistochemical staining (IHC) requires specific antibodies; however, testing inaccuracy and discrepancy among results from studies employing different antibodies has been a major issue.^3^,^10^–^13^ Accordingly, continued investigation of such tests is required.

In this work we studied three antibodies, which are components of different IHC-based HER2 tests. We mapped their epitopes in the HER2 protein and subsequently studied the antibodies’ specificity towards the relevant part of HER2 and homologous parts of HER1, HER3 and HER4. This was conducted in three different immunochemical settings: first, antibody specificity was investigated by staining of formalin-fixed, paraffin-embedded (FFPE) Chinese hamster ovary (CHO) cells transfected with the intracellular domain of HER 1–4, respectively. Secondly, the ability of the antibodies to bind HER1, HER2 and HER4 peptides was tested in a competitive enzyme-linked immunosorbent assay (ELISA). Thirdly, immunoblotting of *Escherichia coli*-expressed fusion proteins consisting of glutathione S-transferase (GST) and part of the intracellular domain of HER1, HER2, HER3 or HER4, respectively, was performed. As all three investigated antibodies are used for determination of HER2 status as an aid in the assessment of patients for whom HER2-targeted therapy is being considered, we expected them to show specificity towards HER2. Hence, our hypothesis states that these antibodies are specific.

Our data confirm that all three investigated antibodies react with HER2 protein. However, here we also show that one antibody cross-reacts with HER4 protein and peptide in both IHC staining of HER4-transfected cells, in competitive ELISA and in immunoblotting. The specificity of this antibody needs further investigation and the potential clinical implications of cross-reactivity should be addressed.

## Materials and methods

### Antibodies

The investigated antibodies are components of different IHC-based HER2 tests: PATHWAY® HER2 (clone 4B5; Ventana Medical Systems Inc., Tucson, AZ, USA), HercepTest™ (Dako Denmark A/S, Glostrup, Denmark) and Oracle® HER2 (clone CB11; Leica Microsystems GmbH, Wetzlar, Germany). The PATHWAY® HER2 and HercepTest™ antibodies were applied in all experiments, whereas two variants of the CB11 antibody were available: for IHC the antibody recommended for staining with the Bond-III was used (Oracle® HER2, clone CB11; Leica Microsystems GmbH), and for ELISA and immunoblotting experiments the corresponding CB11 antibody concentrate was employed (Novocastra NCL-CB11; Leica Microsystems A/S, Ballerup, Denmark). All three antibodies are reported to bind the intracellular region of HER2 near the C-terminal end of the protein (HercepTest™ package insert; Powell *et al.*^10^; Corbett *et al.*^14^).

### Epitope Mapping and Alignment of HER Proteins

The binding sequence in HER2 for each of the three antibodies was delineated using the alanine scanning method.^15^ Briefly, peptides are synthesized substituting an alanine at each position in the peptide, and binding of each antibody to the synthetic peptides is investigated using ELISA (Pepscan Presto BV, Lelystad, the Netherlands).

The region in HER2 harbouring epitopes recognized by the antibodies was aligned with the corresponding regions in HER1, HER3 and HER4.

### Immunohistochemistry

Formalin-fixed, paraffin-embedded cell blocks were made containing CHO K1 cells transfected with the intracellular domain of HER1, HER2, HER3 or HER4, respectively. For construction of plasmids polymerase chain reaction (PCR) was performed using cDNA from human cell lines or commercially available RNA [for HER1 the A431 cell line; for HER2 the SK-BR-3 cell line; for HER3 the MCF-7 cell line; and for HER4 human muscle mRNA (Invitrogen A/S, Taastrup, Denmark)]. Primers were selected to amplify the coding sequences of the proteins’ intracellular domains. PCR products were purified using the MinElute PCR purification kit (Qiagen, Copenhagen, Denmark); sequences were verified by sequencing (Eurofins MWG Operon, Ebersberg, Germany). The chosen vector (pDisplay; Invitrogen A/S) directs the protein into the membrane of the host cell line CHO K1 and harbours a specific tag for determination of transfection efficiency. The purified PCR products were cloned into this vector, transformed into TOP10-competent *E. coli* cells, and plasmids were purified by an EndoFree Plasmid Maxi Kit (Qiagen). CHO K1 cells were transfected with one of the four plasmids, respectively, by incubation with Lipofectamine™ LTX (Invitrogen A/S) for 26 h. Cells were harvested with trypsin, washed in phosphate-buffered saline (PBS) and cell pellets were mixed with 2% agar and transferred to a plastic pipette for construction of cell straws. Cell straws were fixated in formalin [10% formalin in Tris-buffered saline (TBS)] for 24 h. The fixated cells were dehydrated in a tissue processor; 2 × 1 h in 70% alcohol, 2 × 1 h in 96% alcohol, 2 × 1 h in 99% alcohol and 2 × 1 h in xylen. Finally, cells were embedded in paraffin overnight.

Immunohistochemical stainings were performed on automated IHC platforms according to the manufacturers’ instructions (PATHWAY® HER2 on BenchMark ULTRA, HercepTest™ on Dako Autostainer and Oracle® HER2 on Bond-III). Each cell pellet was included twice on each slide and two separate slides were stained per run. Each run was repeated on three independent occasions.

### ELISA

**Figure 1:**
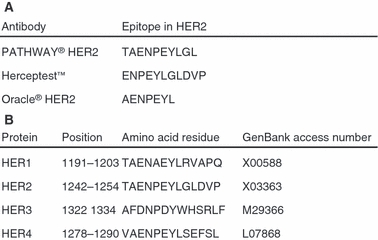
Amino acids in human epidermal growth factor receptor 2 (HER2) that constitute epitopes recognized by each antibody (**A**) and alignment of part of the intracellular domains of HER1, HER2, HER3 and HER4 (**B**).

Synthetic peptides (PolyPeptide Group, Strasbourg, France) were used in ELISA experiments (Figure 1B). The HER2 peptide corresponded to the part of the intracellular domain containing the epitopes (amino acids 1242–1254). Peptides representing HER1 (amino acids 1191–1203), HER3 (amino acids 1322–1334) and HER4 (amino acids 1278–1290) were synthesized to cover the region homologous to HER2. The HER3 peptide could not be employed in ELISA due to unspecific binding of the peptide to the microtitre plate. Accordingly, the ability of the HER3 peptide to compete with antibody binding to HER2 in ELISA was not investigated.

For the competitive ELISA, microtitre plates were coated overnight (4°C) with HER2 peptide (1 μg/ml in carbonate buffer, pH 9.6). Next, plates were incubated (1 h, 30°C) with HER2 antibody together with increasing concentrations of HER1, HER2 or HER4 peptides (concentration range, 0–10 μg/ml). After washing, remaining antibody was detected using relevant secondary alkaline phosphatase-conjugated antibodies (Dako A/S) and incubation with *p*-nitrophenyl phosphate substrate. After 1 h, colour development was quantified at 405 nm in an automatic plate reader (BioTek Instruments Inc., Winooski, VT, USA). Optical density (OD) values were normalized to control wells with coating peptide and antibody only (100% signal). Prior to performing the ELISA, determination of appropriate primary antibody concentrations was performed by titration of the three antibodies individually against coated antigen; the concentrations of the antibodies were selected to give OD values approximately in the middle of the dynamic range. ELISA experiments were performed independently three times.

### Immunoblotting

Protein fragments representing part of the intracellular domain of HER1, HER2, HER3 and HER4 fused to GST were constructed. Briefly, domain-encoding sequences were amplified using standard PCR techniques; sequences were verified by sequencing (Eurofins MWG Operon, Ebersberg, Germany) and subcloned into the pIVEX-GST expression vector (Roche Diagnostics A/S, Hvidovre, Denmark) downstream of the *GST* gene; the construct also includes a C-terminal hexa-histidine affinity tag. Fusion proteins were expressed in *E. coli* and purified as soluble or insoluble aggregates using nickel chelating affinity chromatography purification techniques. The concentration of GST-fusion protein and protein identity was evaluated using sodium dodecyl sulphate-polyacrylamide gel electrophoresis (SDS-PAGE) Coomassie staining and anti-GST immunoblotting. Equal amounts of specific proteins (GST–HER1, –HER2, –HER3 and –HER4) were loaded onto the gels; this was confirmed using a GST-specific antibody (1:30 000; GE Healthcare Europe GmbH, Brondby, Denmark).

Gel electrophoresis was performed using an XCell *SureLock*™ Mini-Cell electrophoresis system with NuPAGE® 4–12% BisTris gels according to the manufacturer's instructions (Invitrogen A/S), followed by immunoblotting using a semi-dry transfer unit (GE Healthcare Europe GmbH) and incubation with primary antibodies and secondary horseradish peroxidase-conjugated antibodies. HER2 antibodies were diluted 1:3000 (PATHWAY® HER2), 1:1000 (HercepTest™) and 1:80 000 (CB11 concentrate). Secondary antibodies (Dako A/S) were diluted 1:3000. Blots were developed using Amersham ECL Advance or ECL Plus Western Blotting Detection Kits (GE Healthcare Europe GmbH), according to the manufacturer's instructions. After development and visualization, blots were stripped (62.5 mm Tris, 2% SDS, 0.7%β-mercaptoethanol), reprobed with HER4 antibody (1:4000, OriGene TA303501-100; Rockville, MD, USA) and developed. Bands were visualized in an UVP Biospectrum Imaging System; the resulting images were inverted in ImageJ for clearer visualization. Experiments were performed independently three times.

## Results

### Epitope Mapping and Alignment of HER Proteins

Figure 1A shows the result of epitope mapping and illustrates amino acid residues in HER2 that constitute epitopes recognized by each of the antibodies. The antibodies bind to overlapping sequences and the recognized epitopes share a sequence of six consecutive amino acids (HER2 amino acids 1244–1249). The Oracle® HER2 antibody binds to the shortest epitope (seven amino acid residues) compared to Herceptest™ and PATHWAY® HER2 antibodies (epitopes of 11 and 10 amino acid residues, respectively). Figure 1B shows the amino acid sequence in HER2 that harbours all three epitopes and the sequences in corresponding parts of HER1, HER3 and HER4.

### Immunohistochemical Staining

**Figure 2:**
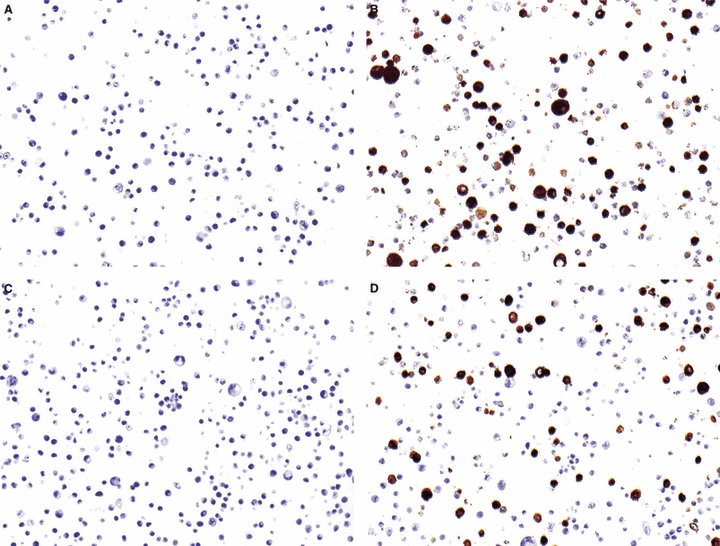
PATHWAY® human epidermal growth factor receptor 2 (HER2) immunostaining of Chinese hamster ovary (CHO) K1 cells transfected with the intracellular domain of HER1 (**A**), HER2 (**B**), HER3 (**C**) or HER4 (**D**). A representative area is shown.

**Figure 3:**
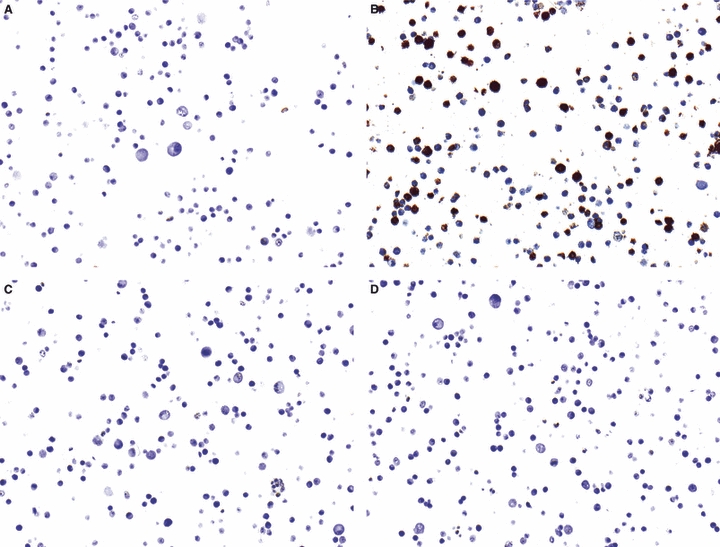
HercepTest™ human epidermal growth factor receptor 2 (HER2) immunostaining of Chinese hamster ovary (CHO) K1 cells transfected with the intracellular domain of HER1 (**A**), HER2 (**B**), HER3 (**C**) or HER4 (**D**). A representative area is shown.

**Figure 4:**
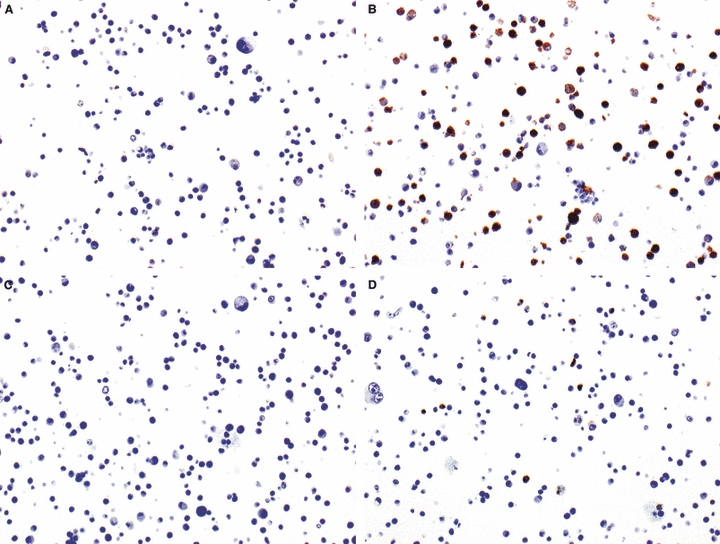
Oracle® human epidermal growth factor receptor 2 (HER2) immunostaining of Chinese hamster ovary (CHO) K1 cells transfected with the intracellular domain of HER1 (**A**), HER2 (**B**), HER3 (**C**) or HER4 (**D**). A representative area is shown.

Figures 2–4 show immunostaining of FFPE CHO K1 cells transfected with the intracellular domain of HER1, HER2, HER3 or HER4. Transfection efficiency for the applied protocol was 10–50%; this was confirmed by staining transfected cells with a tag-specific antibody (data not shown). Figure 2 shows cells stained with PATHWAY® HER2; this antibody stained HER2-transfected cells and cells transfected with the intracellular domain of HER4. With HercepTest™ (Figure 3), only cells transfected with the intracellular domain of HER2 were stained. Finally, the Oracle® HER2 antibody stained HER2 transfected cells and weakly stained few HER4 transfected cells (Figure 4). Representative areas are shown for all immunostainings.

### Competitive ELISA

**Figure 5:**
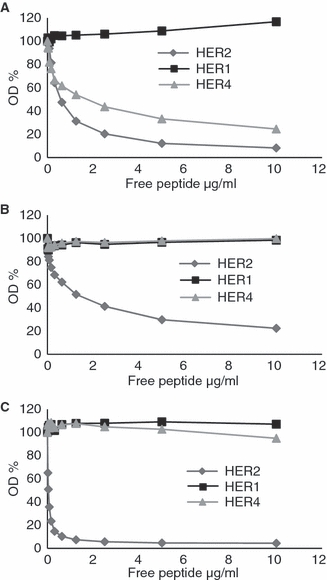
Results of competitive enzyme-linked immunosorbent assay (ELISA). Human epidermal growth factor receptor 1 (HER1), HER2 and HER4 peptides were added and competition investigated for the PATHWAY® HER2 antibody (**A**), the HercepTest™ antibody (**B**) and the CB11 antibody concentrate (**C**). The figure shows the result of one experiment representative of three independent ones.

Antibody dilution experiments showed good ELISA performance (50% OD_max_) for the PATHWAY® HER2 antibody at 1:40 dilution, for the HercepTest™ antibody at 1:20 dilution, and for the CB11 antibody concentrate at 1:1600 dilution. Figure 5A shows the result of a competitive ELISA employing the PATHWAY® HER2 antibody; here, addition of HER2 and HER4 peptides, respectively, competed with coated HER2 peptide for antibody binding, whereas addition of HER1 peptide did not. A similar competitive ELISA (Figure 5B) showed that only added HER2, and not HER1 or HER4, competed with coated peptide for the HercepTest™ antibody's binding. Similarly, only added HER2 competed with coated HER2 peptide for binding to the CB11 antibody (Figure 5C).

### Immunoblotting

**Figure 6:**
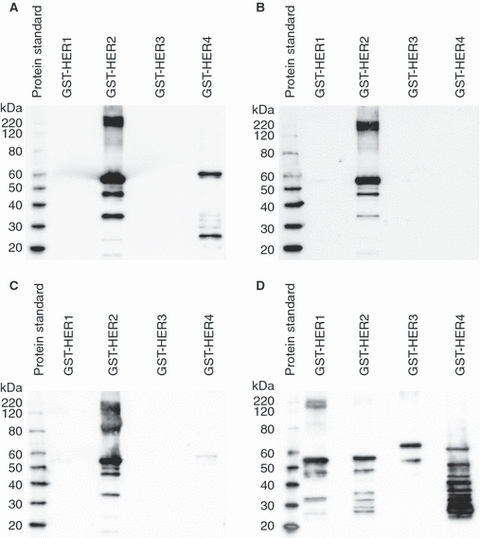
Immunoblots of glutathione S-transferase (GST)–human epidermal growth factor receptor (HER) fusion proteins. Identical blots were developed using the PATHWAY® HER2 antibody (**A**), the HercepTest™ antibody (**B**) and the CB11 antibody concentrate (**C**). **D**, The loading control (anti-GST). The figure shows the result of one experiment representative of three independent ones.

**Figure 7:**
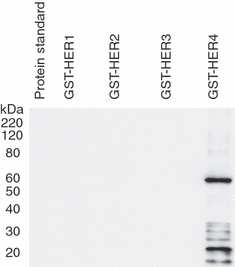
Visualization of glutathione S-transferase (GST)–human epidermal growth factor receptor 4 (HER4) after stripping and reprobing of the blot initially developed using the PATHWAY® HER2 antibody.

Figures 6A–D and 7 show the results of immunoblotting experiments. The PATHWAY® HER2 antibody detected both GST–HER2 and GST–HER4 (Figure 6A); the identity of GST–HER4 was confirmed by stripping and reprobing the blot using a HER4 antibody (Figure 7). HercepTest™ (Figure 6B) and CB11 (Figure 6C) antibodies did not cross-react with GST–HER4, although vague staining of GST–HER4 could be achieved with the CB11 antibody after extended exposure time. The control blot (Figure 6D) shows that equal amounts of GST-fusion proteins were loaded.

## Discussion

In this study we investigated the specificity of three pharmacodiagnostic HER2 antibodies towards fusion proteins and synthetic peptides homologous to proteins of the HER family. All three antibodies reacted with HER2 proteins and peptides. One antibody (PATHWAY® HER2) did not bind HER1 or HER3, but clearly cross-reacted with HER4 proteins and peptides in all our experiments: in IHC, HER4-transfected cells were intensely stained with the PATHWAY® HER2 antibody; a synthetic peptide corresponding to part of the HER4 intracellular domain competed with antibody binding to HER2 in ELISA; and a GST–HER4 band was detected in immunoblotting. Thus, this antibody reacted with HER4 across three different experimental techniques. The second antibody (from HercepTest™) did not cross-react with HER1, HER3 or HER4, while the third antibody (Oracle® HER2) weakly stained a small number of cells transfected with the intracellular domain of HER4 and was able to detect a GST–HER4 fusion protein when applied in immunoblotting. The HER4 synthetic peptide did not show competition with binding of the CB11 antibody to HER2 in ELISA.

Our immunohistochemical stainings were performed strictly according to the manufacturers’ instructions on the recommended equipment; hence the observed reactivity to HER2, and for the PATHWAY® HER2 antibody also to HER4, can be expected to occur when using these kits in a routine setting. Our ELISA included positive as well as negative controls for comparison and monitoring of assay performance. Immunoblots were performed similarly for all antibodies and the identity of the HER4 protein to which the PATHWAY® HER2 antibody bound was confirmed by stripping and reprobing the membrane.

The HER2 protein shows sequence homology to other HER family members in the C-terminal part (Figure 1B). HER1 and HER4 sequences show most overlap with HER2 in this region of the protein; the alignment of intracellular regions of the proteins reveals that over a sequence of seven amino acids, HER2 and HER4 are 100% homologous whereas HER2 and HER1 proteins also share wide sequence homology but only an uninterrupted, homologous sequence of four amino acids. HER3 shows the least homology to HER2; alignment of HER2 and HER3 shows homology at three positions (HER3 residues 1325, 1326 and 1328). Based on these alignments and on the epitope mapping, extensive cross-reactivity is theoretically possible as all three antibodies recognize partly overlapping epitopes, and HER2 has sequence homology with both HER1 and HER4. However, we only consistently found cross-reactivity for the PATHWAY® HER2 antibody and only to HER4. Our results are not in keeping with previously published data suggesting that the PATHWAY® HER2 antibody does not cross-react with other members of the HER family:^10^ in an immunoblot of various cell lysates, Powell *et al.* showed that the PATHWAY® HER2 antibody stained only HER2 and not HER1, HER3 or HER4. This report, however, neither demonstrated how much HER protein was loaded nor adjusted loading to ensure equal amounts of HER1, HER2, HER3 and HER4 protein. In the present study, we loaded equal amounts of HER–GST fusion proteins (Figure 6D) and also confirmed the identity of the cross-reacting protein with a HER4 specific antibody (Figure 7). We observed cross-reactivity for the PATHWAY® HER2 antibody across three different experimental platforms and in particular, we found staining of HER4-transfected cells. The use of FFPE-transfected cells probably reflects actual HER family members’ epitopes in FFPE material. Additionally, the results of the epitope mapping (Figure 1A) demonstrated that all three antibodies recognize linear epitopes; consequently, these are likely to be similar in our peptides and fusion proteins and in full-length native protein.

Weak staining of HER4-transfected cells was also observed for the Oracle® HER2 antibody; however, as this staining was not intense and only present in a small fraction of cells we speculate that it is due to high expression levels of HER4 protein in some transfected cells, and that this may not occur in clinical samples. The Oracle® Bond HER2 immunohistochemical staining system employs affinity-purified CB11 antibody,^16^ whereas the CB11 concentrate used for ELISA and immunoblotting is not purified. However, although unpurified, the CB11 concentrate was specific in ELISA and only showed a very faint band in immunoblots.

Our study indicates that the PATHWAY® HER2 antibody can bind HER4 homologous protein and peptide in three different experimental settings. However, as described, we have not detected native human HER proteins in this study. Thus, it remains to be shown whether staining of breast cancer tissue sections with this particular antibody leads to detection of HER4 protein. If so, it should be investigated whether the potential detection of HER4 protein would have any clinical implications. It may be possible that patients believed to be HER2-positive would receive HER2-targeted therapy while in fact being only HER4-positive. Similarly, HER2 low-expressing tumours (1+) could be classified erroneously as 2+ or 3+ due to detection of HER4 together with HER2. A study of 130 breast carcinomas did yield more 2+ and 3+ cases when using the PATHWAY® HER2 antibody than when employing the HercepTest™ antibody, although a very high concordance between PATHWAY® HER2 IHC and fluorescence *in-situ* hybridization (FISH) was found.^13^ Conversely, if HER4 is expressed mainly together with high levels of HER2, the simultaneous detection of both proteins would have no clinical implications in the assignment or not of HER2-targeted therapy. Expression of HER4 protein in breast cancer tissue has been demonstrated; however, uncertainty about the level and extent of expression persists. Examining only the presence or absence of staining, a study of 191 breast cancer tissue samples revealed HER4 protein expression in 37.2% of the samples; this study also reported that 71% of HER2 overexpressing cases coexpressed HER4.^7^ Another group reported HER4 protein expression in 69.5% of 220 breast cancer tissue samples but demonstrated that HER4 was rarely coexpressed with other members of the HER family.^6^ A third study found HER4 staining in only six of 54 (11%) breast tumours.^8^ Thus, despite lack of consistency across studies, HER4 protein is expressed in breast cancer tissue, and two studies^6^,^7^ suggest that HER4 expression may indeed be present without coexpression of HER2. Thus, a false-positive HER2 signal could be possible if using a HER2 antibody which cross-reacts with HER4.

In conclusion, our study shows that one pharmacodiagnostic antibody can bind HER4 protein and peptide in IHC, ELISA and immunoblots, suggesting that it could also bind to the intracellular domain of HER4 in clinical breast cancer samples. It should be investigated whether such cross-reactivity occurs in breast cancer tissue and whether potential detection of HER4 protein could have implications for selection of treatment for breast cancer patients.

## Competing interests

Nils Brünner is medical adviser to Dako A/S. Hans Christian Pedersen and Sussie Steen Jensen are employed at Dako Denmark A/S. Reagents were paid in part by Dako Denmark A/S.

## Abbreviations

CHO: Chinese hamster ovary
EGFR: epidermal growth factor receptor
ELISA: enzyme-linked immunosorbent assay
FFPE: formalin-fixed, paraffin-embedded
GST: glutathione S-transferase
HER2: human epidermal growth factor receptor 2
PCR: polymerase chain reaction
